# Supplementary material for: Cough Characteristics and Their Association Patterns According to Cough Etiology: A Network Analysis
Source: J Clin Med. 2023 Aug 18;12(16):5383. doi: 10.3390/jcm12165383 (PMC10455312; doi:10.3390/jcm12165383)
Supplement: Supplementary file 1 [file jcm-12-05383-s001.zip › jcm-2555220-supplementary.pdf]

Supplementary Materials

Cough Characteristics and Their Association Patterns According to Cough Etiology: A Network Analysis

Jieun Kang [1], Ji-Yong Moon [2], Deog Kyeom Kim [3], Jin Woo Kim [4], Seung Hun Jang [5] and Hyeon-Kyoung Koo [1,*]

1 Division of Pulmonary and Critical Care Medicine, Department of Internal Medicine, Ilsan Paik Hospital, Inje University College of Medicine, Goyang 10380, Republic of Korea; realodette@gmail.com

2 Department of Internal Medicine, Hanyang University College of Medicine, Guri 11923, Republic of Korea; respiry@gmail.com

3 Division of Pulmonary and Critical Care Medicine, Department of Internal Medicine, Seoul Metropolitan Government-Seoul National University Boramae Medical Center, Seoul National University College of Medicine, Seoul 07061, Republic of Korea; kimdkmd@snu.ac.kr

4 Division of Pulmonary and Critical Care Medicine, Department of Internal Medicine, Uijeongbu St. Mary's Hospital, College of Medicine, The Catholic University of Korea, Uijeongbu 11765, Republic of Korea; medkjw@catholic.ac.kr

5 Division of Pulmonary, Allergy, and Critical Care Medicine, Department of Medicine, Hallym University Sacred Heart Hospital, Hallym University College of Medicine, Anyang 14068, Republic of Korea; chestor@hallym.or.kr

* Correspondence: gusrud9@yahoo.co.kr; Tel.: +82-31-910-7215

Figure S1. Histograms for age distribution according to the cause of chronic cough.

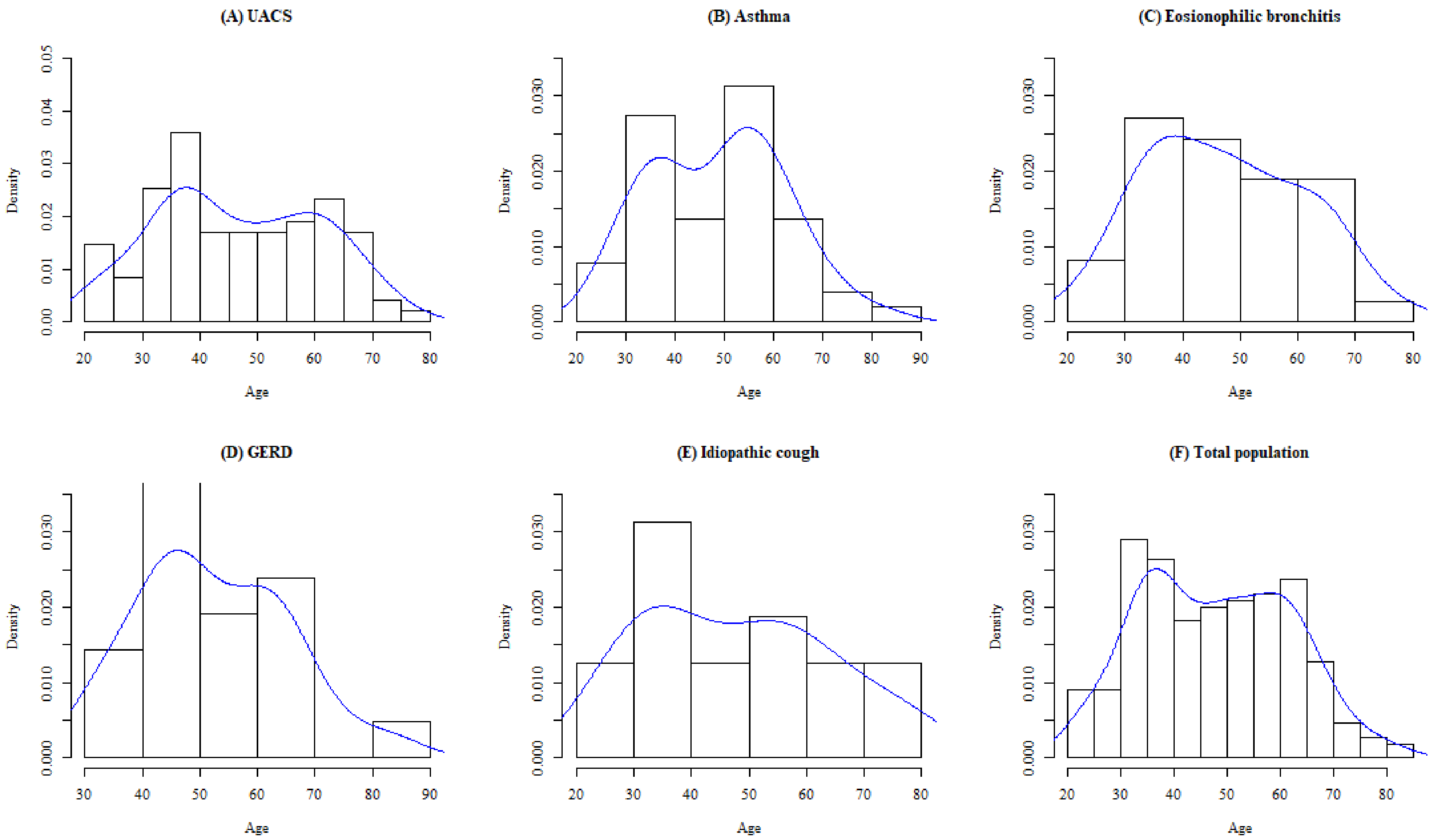

Abbreviation: UACS, Upper Airway Cough Syndrome; GERD, Gastroesophageal reflux disease.

Figure S2. Association between age and cough severity using K-LCQ scores according to cough etiology.

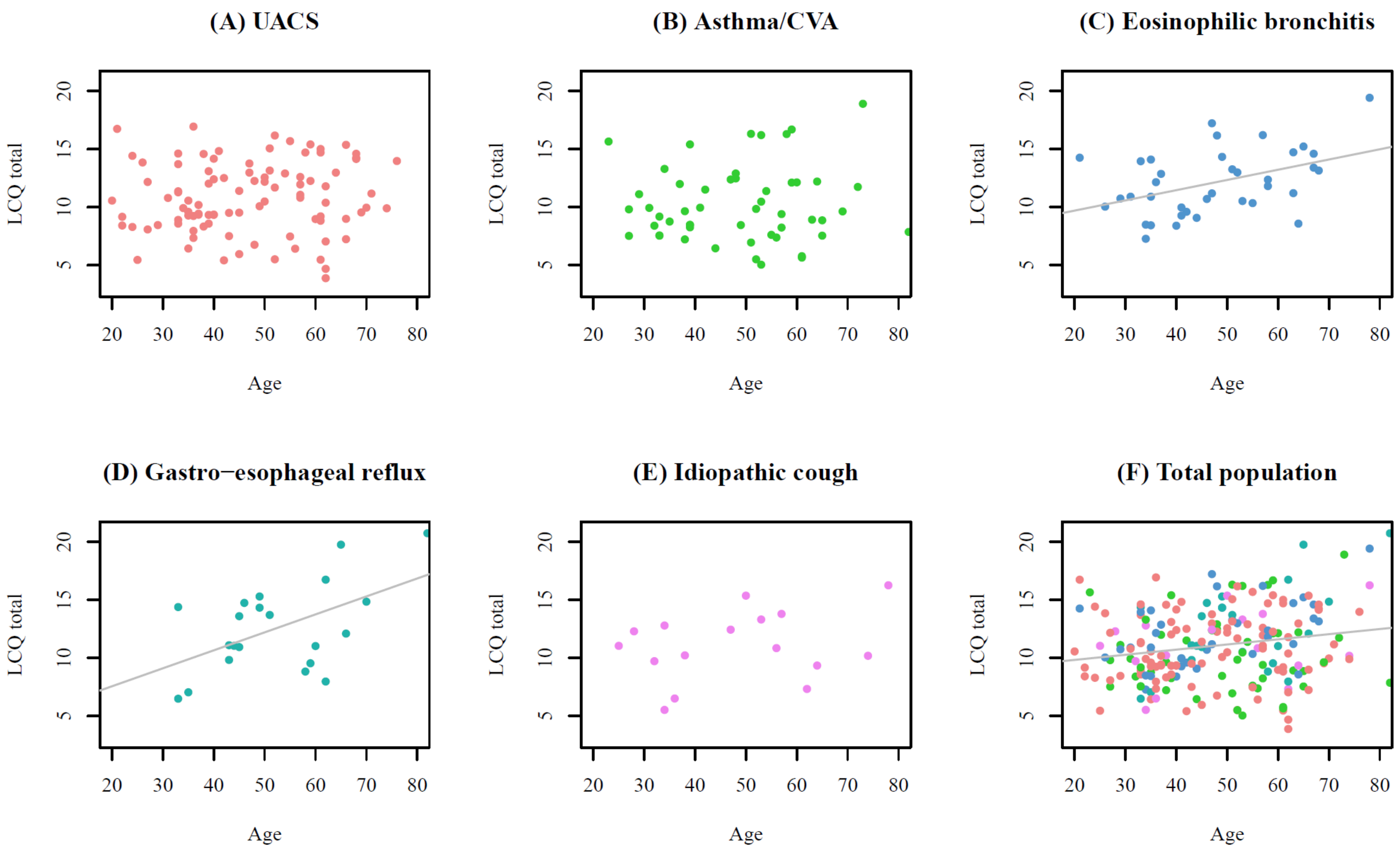

Abbreviation: K-LCQ, Korean version of Leicester Cough Questionnaire; UACS, Upper Airway Cough Syndrome; CVA, cough variant asthma.

Figure S3. Association between age and cough severity using NRS scores according to cough etiology.

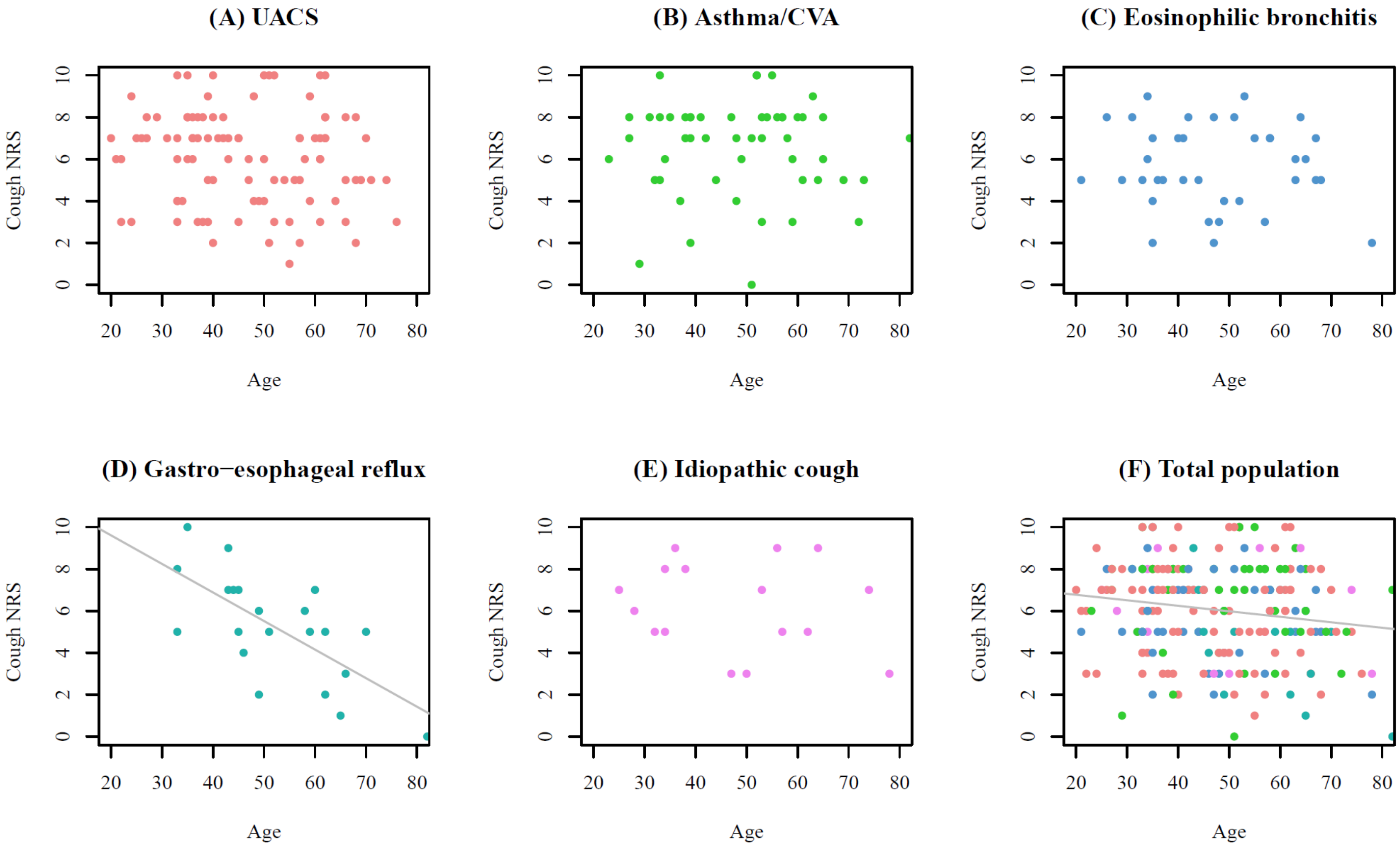

Abbreviation: NRS, numeric rating scale; UACS, Upper Airway Cough Syndrome; CVA, cough variant asthma.

Table S1. Correlation matrix showing Pearson correlation coefficients in each cough etiology.

(A) Upper airway cough syndrome

	Age	Female	COAT1	COAT2	COAT3	COAT4	COAT5
**Age**	1.000	0.373*	-0.134	-0.232*	0.071	-0.078	0.014
**Female sex**		1.000	-0.039	-0.188	0.060	0.008	-0.004
**COAT1**			1.000	0.618*	0.501*	0.603*	0.446*
**COAT2**				1.000	0.504*	0.681*	0.416*
**COAT3**					1.000	0.581*	0.242*
**COAT4**						1.000	0.425*
**COAT5**							1.000

(B) Asthma

	Age	Female	COAT1	COAT2	COAT3	COAT4	COAT5
**Age**	1.000	0.163	-0.189	-0.146	-0.003	-0.033	-0.011
**Female sex**		1.000	0.026	0.123	0.216	0.318*	-0.069
**COAT1**			1.000	0.651*	0.628*	0.461*	0.153
**COAT2**				1.000	0.593*	0.724*	0.230
**COAT3**					1.000	0.522*	0.165
**COAT4**						1.000	0.287*
**COAT5**							1.000

(C) Eosinophilic bronchitis

	Age	Female	COAT1	COAT2	COAT3	COAT4	COAT5
**Age**	1.000	0.010	-0.282	-0.424*	-0.161	-0.391*	-0.370*
**Female sex**		1.000	0.274	0.245	0.316	0.455*	0.342*
**COAT1**			1.000	0.563*	0.382*	0.530*	0.374*
**COAT2**				1.000	0.362*	0.608*	0.545*
**COAT3**					1.000	0.494*	0.068
**COAT4**						1.000	0.620*
**COAT5**							1.000

(D) Gastroesophageal reflux

	Age	Female	COAT1	COAT2	COAT3	COAT4	COAT5
**Age**	1.000	0.281	-0.464*	-0.492*	-0.277	-0.351	-0.498*
**Female sex**		1.000	-0.243	-0.376	-0.406	-0.080	-0.098
**COAT1**			1.000	0.828*	0.380	0.488*	0.549*
**COAT2**				1.000	0.372	0.465*	0.575*
**COAT3**					1.000	0.732*	0.338
**COAT4**						1.000	0.596*
**COAT5**							1.000

(E) Idiopathic cough

	Age	Female	COAT1	COAT2	COAT3	COAT4	COAT5
**Age**	1.000	0.205	-0.333	-0.330	0.139	-0.233	-0.293
**Female sex**		1.000	-0.796*	-0.563*	-0.490	-0.340	-0.602*
**COAT1**			1.000	0.757*	0.596*	0.658*	0.529*
**COAT2**				1.000	0.662*	0.749*	0.610*
**COAT3**					1.000	0.782*	0.236
**COAT4**						1.000	0.305
**COAT5**							1.000

* Indicates statistical significance ($p < 0.05$).
